# Systemic immune-inflammation index predicts the clinical outcome in patients with nasopharyngeal carcinoma: a propensity score-matched analysis

**DOI:** 10.18632/oncotarget.19796

**Published:** 2017-08-02

**Authors:** Wenjie Jiang, Yuan Chen, Jin Huang, Dan Xi, Jun Chen, Yingjie Shao, Guoping Xu, Wenming Ying, Jun Wei, Junjun Chen, Zhonghua Ning, Wendong Gu, Honglei Pei

**Affiliations:** ^1^ Department of Radiation Oncology, The Third Affiliated Hospital of Soochow University, Changzhou 213003, P.R. China; ^2^ Department of Respiratory, The Seventh People’s Hospital of Changzhou, Changzhou 213000, P.R. China

**Keywords:** SII, nasopharyngeal carcinoma, prognosis, PSM

## Abstract

Systemic immune-inflammation index (SII), based on peripheral lymphocyte, neutrophil, and platelet counts, was recently investigated as a prognostic marker in several tumors. However, SII has not been reported in nasopharyngeal carcinoma (NPC). We evaluated the prognostic value of the SII in 327 patients with NPC. Univariate and multivariate analyses were calculated by the Cox proportional hazards regression model. The time-dependent receiver operating characteristics (ROC) curve was used to compare the discrimination ability for OS. PSM (propensity score matching) was carried out to imbalance the baseline characteristics. Our results showed that SII, PLR, NLR and MLR were all associated with OS in NPC patients in the Kaplan-Meier survival analysis. SII (HR: 2.26; 95% CI: 1.40-3.66; P=0.001), NLR (HR: 1.66; 95% CI: 1.08-2.53; P=0.020), and MLR (HR: 1.99; 95% CI: 1.17-3.39; P=0.011) were identified to be the independent prognostic factors. The AUC for SII was bigger than NLR, PLR and MLR for predicting survival in patients with NPC in 3 or 5-years. In the PSM analysis, SII remained an independent predictor for OS in NPC patients (HR=2.08, CI 1.22-3.55, P=0.007). SII is a novel, simple and inexpensive prognostic predictor for patients with NPC. The prognostic value of SII is superior to PLR, NLR and MLR.

## INTRODUCTION

The incidence of nasopharyngeal carcinoma (NPC) has obvious geographic distributions. The high incidence of NPC occurs in southeast of China, Hong Kong and Singapore. There were 60,600 new cases and 34,100 deaths of NPC per year in China [1]. Radiotherapy is the preferred treatment for NPC. Although multidisciplinary treatment based on radiotherapy has achieved good results, local failure and distant metastasis are still more common [2]. Patients with recurrence or distant metastasis had poor prognosis, and five-year overall survival (OS) is less than 30% [3]. Currently, the gold standard for predicting the prognosis of NPC patients is the American Joint Committee on Cancer tumor-node-metastasis (AJCC TNM) staging system. However, NPC patients at the same TNM stage and received similar therapy usually had different outcomes [4, 5]. Therefore, it is important to explore the biomarkers which can improve prognostic prediction of NPC.

Since the first reported by Virchow in 1863 [6], there have been accumulating evidences supporting that inflammation contributes to tumor growth, progression and metastasis [7]. Recently, the systemic inflammatory response biomarkers such as circulating immune cells have been found to be independent markers of prognosis in a variety of cancers. Circulating immune cells mainly included neutrophil, platelet, lymphocyte and monocyte which derived from the peripheral blood. Systemic inflammatory biomarkers such as platelet lymphocyte ratio (PLR), neutrophil lymphocyte ratio (NLR) and monocyte lymphocyte ratio (MLR) have emerged as prognostic markers in a variety of cancer, including NPC [8–10]. These markers only integrate two circulating immune cells. Recently, systemic immune-inflammation index (SII), based on three circulating immune cells (peripheral lymphocyte, neutrophil, and platelet), has proved to be a novel prognostic marker in hepatocellular carcinoma [11–13], esophageal cancer [14], colorectal cancer [15], gastric cancer [16], small cell lung cancer [17] and renal cell cancer [18]. However, the prognostic value of SII has not been reported in NPC. In our study, we first reported the prognostic value of SII in patients with NPC. We also evaluated whether SII has more advantages to predict prognosis of NPC than other systemic inflammatory biomarkers. To increase statistical power and to further elaborate on the possible prognostic impact of SII, both Cox’s proportional hazards model analysis as well as propensity score matching (PSM) were applied.

## RESULTS

### Baseline clinical and characteristics

Finally, a total of 327 patients were enrolled in this study. There were 243 males (74.3%) and 84 females (25.7%) with age ranging from 20 to 80 years (median 50 years). Among them, 87 patients’ body mass index (BMI) were over 25. According to the TNM stage, there were 12 patients at stage I, 54 patients at stage II, 146 patients at stage III and 115 patients at stage IV. The 3- and 5-year overall survival (OS) rates were 76.3% and 65.7%, respectively. The association between SII, PLR, NLR, MLR and clinicopathological features are shown in Table 1, 2. Patients with SII>403 were more likely to be BMI>25 (*P*=0.002), advanced T stage (*P*=0.013) and advanced TNM stage (*P*=0.031) (Table 1). We also explored the association between SII, PLR, NLR and MLR (Table 2). It was found that SII was associated with other inflammation-based prognostic indexes (all *P*<0.001).

**Table 1:** Baseline characteristics for patients with SII≤403 versus SII>403 before and after propensity matching

Clinical parameter	Unmatched (complete) dataset					Matched (1:1) dataset				
	SII≤403 (123)	SII>403 (204)	χ^2^	*P*	S.D	SII≤403 (108)	SII>403 (108)	χ^2^	*P*	S.D
Sex			0.46	0.498				0.41	0.519	
Male	94	149			0.08	85	81			0.09
Female	29	55			0.08	23	27			0.09
Age mean(SD)	54(11)	52(12)			0.17	53(12)	52(12)			0.08
BMI			10.06	0.002^*^				0.33	0.564	
≤25	78	162			0.70	70	74			0.08
>25	45	42			0.70	38	34			0.08
T stage			10.78	0.013^*^				1.54	0.674	
T1	34	27			0.36	23	20			0.07
T2	29	57			0.10	27	28			0.01
T3	38	71			0.08	35	30			0.10
T4	22	49			0.15	23	30			0.15
N stage			6.51	0.089				1.77	0.625	
N0	20	15			0.28	9	8			0.03
N1	29	57			0.10	29	35			0.12
N2	55	99			0.08	51	52			0.02
N3	19	33			0.01	19	13			0.16
AJCC stage			8.85	0.031^*^				0.00	1.000	
I	9	3			0.29	3	3			0
II	23	31			0.09	21	21			0
III	53	93			0.05	47	47			0
IV	38	77			0.15	37	37			0
chemotherapy			3.26	0.071				0.43	0.513	
No	32	36			0.20	26	22			0.09
Yes	91	168			0.20	82	86			0.09
IMRT			1.12	0.291				0.70	0.402	
No	79	119			0.12	69	63			0.11
Yes	44	85			0.12	39	45			0.11

SD: standard deviation; BMI: body mass index; AJCC: American Joint Committee on Cancer; IMRT: intensity-modulated radiotherapy; SII: systemic immune-inflammation index; S.D: standardized difference.

**Table 2:** Relationship between NLR, PLR or MLR and clinicopathological characteristics of patients with nasopharyngeal carcinoma

Clinical parameter	NLR				PLR				MLR			
	≤2.26 (158)	>2.26 (169)	χ^2^	*P*	≤112 (141)	>112 (186)	χ^2^	*P*	≤0.25 (97)	>0.25 (230)	χ^2^	*P*
Sex			1.88	0.170			0.93	0.334			0.33	0.564
Male	112	131			101	142			70	173		
Female	46	38			40	44			27	57		
Age			0.05	0.828			1.31	0.288			0.30	0.584
<50	58	64			48	74			34	88		
≥50	100	105			93	112			63	142		
BMI			2.23	0.135			7.02	0.008			3.88	0.049
≤25	110	130			93	147			64	176		
>25	48	39			48	39			33	54		
T stage			1.06	0.787			0.70	0.874			5.98	0.113
T1	30	31			28	33			16	45		
T2	41	45			35	51			31	55		
T3	56	53			49	60			36	73		
T4	31	40			29	42			14	57		
N stage			4.46	0.216			4.69	0.196			2.89	0.409
N0	16	19			19	16			13	22		
N1	34	52			30	56			23	63		
N2	79	75			67	87			42	112		
N3	29	23			25	27			19	33		
7^th^ AJCC TNM stage			2.75	0.432			4.44	0.217			0.491	0.921
I	5	7			7	5			3	9		
II	21	33			17	37			17	37		
III	75	71			66	80			45	101		
IV	57	58			51	64			32	83		
chemotherapy			0.26	0.613			0.21	0.644			0.298	0.585
No	31	37			31	37			22	46		
Yes	127	132			110	149			75	184		
IMRT			0.69	0.406			0.69	0.408			0.098	0.754
No	92	106			89	109			60	138		
Yes	66	63			52	77			37	92		
SII			39.66	<0.001^*^			98.08	<0.001^*^			43.91	<0.001^*^
≤403	87	36			96	27			63	60		
>403	71	133			45	159			34	170		
PLR			155.87	<0.001^*^							34.92	<0.001^*^
≤112	124	17							66	75		
>112	34	152							31	155		
NLR			-	-			155.87	<0.001^*^			33.18	<0.001^*^
≤2.26	-	-			124	34			71	87		
>2.26	-	-			17	152			26	143		
MLR			33.18	<0.001^*^			34.92	<0.001^*^			-	-
≤0.25	71	26			66	31			-	-		
>0.25	87	143			75	155			-	-		

BMI: body mass index; AJCC: American Joint Committee on Cancer; IMRT: intensity-modulated radiotherapy; SII: systemic immune-inflammation index; PLR: platelet lymphocyte ratio; NLR: neutrophil lymphocyte ratio; MLR: monocyte lymphocyte ratio.

### The prognostic significance of SII, NLR, PLR and MLR

**Figure 1:**
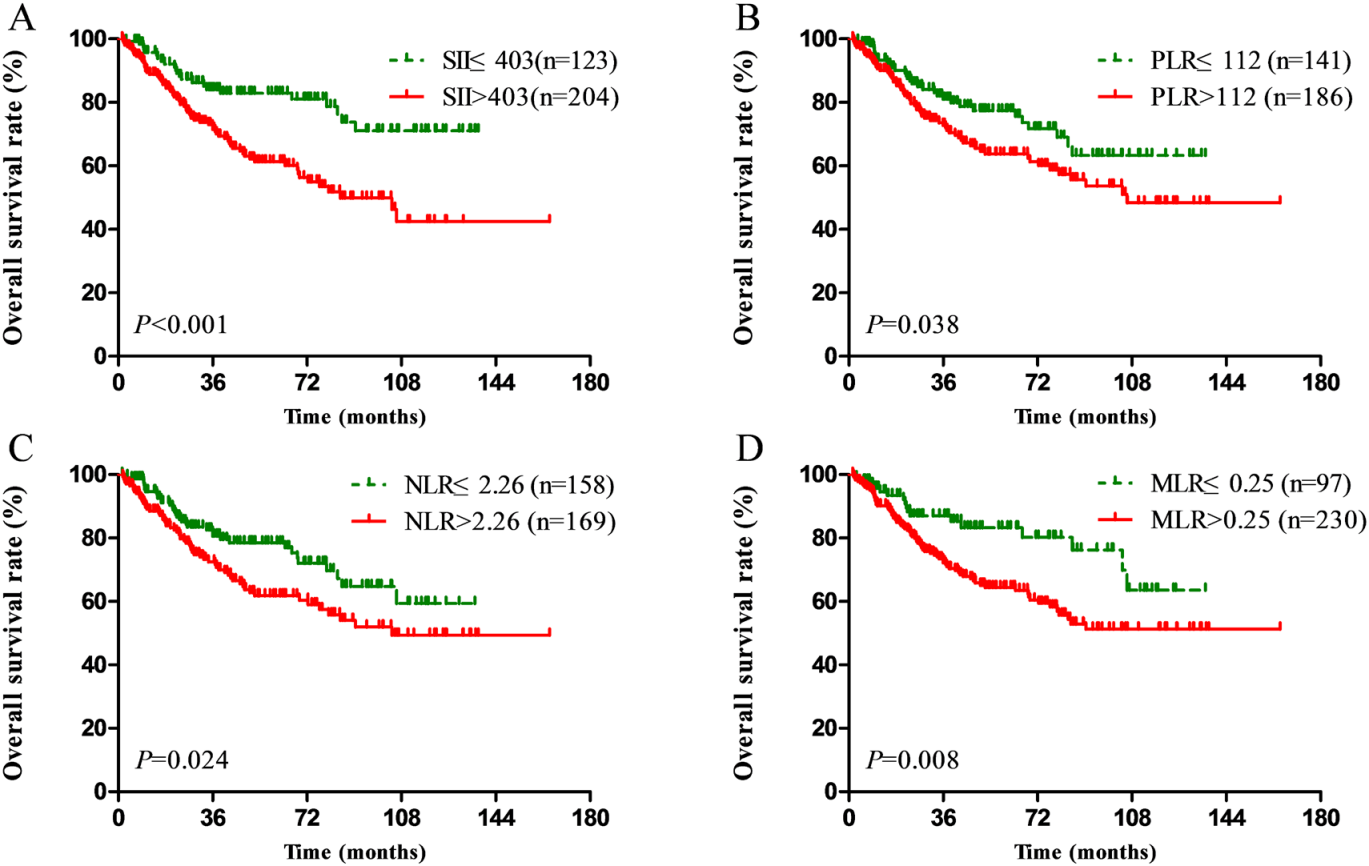
Kaplan–Meier survival curves for patients stratified based on **(A)** SII, **(B)** PLR, **(C)** NLR and **(D)** MLR in unmatched complete datasets.

Compared with a lower SII (≤403), a higher SII (>403) was associated with significant worse OS in NPC patients (*P*<0.001, Figure 1A). High PLR, NLR and MLR scores were also associated with poor OS (*P*=0.038, *P*=0.024, *P*=0.008, respectively) (Figure 1B, 1C, 1D). In the univariate analysis, sex, age, T stage, N stage, SII, PLR, NLR and MLR were identified as the significant prognostic indexes (Table 3). We found that SII, PLR, NLR and MLR were high correlation. So, four separate multivariate models (SII, PLR, NLR and MLR) were run to avoid problems with the presence of multicollinearity. In the multivariate analysis, SII (HR: 2.26; 95% CI: 1.40-3.66; *P*=0.001), NLR (HR: 1.66; 95% CI: 1.08-2.53; *P*=0.020), and MLR (HR: 1.99; 95% CI: 1.17-3.39; *P*=0.011) were identified to be the independent prognostic factors, after adjustment for other characteristics (Table 3). Among the four inflammation-based prognostic indexes, only PLR was not an independent risk factor for OS (*P*>0.05) (Table 3).

**Table 3:** Univariate and multivariate cox regression analyses for overall survival in patients with nasopharyngeal carcinoma (unmatched complete datasets)

Variables	Univariate analysis		Multivariate analysis	
	HR (95%CI)	*P* value	HR (95%CI)	*P* value
Sex				
Female vs. Male	0.39 (0.22-0.69)	0.001^*^	0.40 (0.23-0.72)	0.002^*^
Age				
≥50 vs. <50	1.63 (1.05-2.51)	0.028^*^	1.85 (1.18-2.88)	0.007^*^
BMI				
>25 vs. ≤25	0.75 (0.46-1.24)	0.264		
T stage		0.009^*^		0.039^*^
T1	Ref.		Ref.	
T2	1.77 (0.87-3.61)	0.114	1.67 (0.81-3.44)	0.167
T3	2.39 (1.21-4.72)	0.012^*^	2.11 (1.06-4.21)	0.034^*^
T4	3.199 (1.57-6.51)	0.001^*^	2.75 (1.34-5.66)	0.006^*^
N stage		0.027^*^		0.031^*^
N0	Ref.		Ref.	
N1	2.34 (0.97-5.64)	0.060	2.24 (0.92-5.44)	0.075
N2	2.83 (1.20-6.65)	0.017	2.92 (1.23-6.94)	0.015^*^
N3	4.07 (1.59-10.41)	0.003	4.04 (1.54-10.61)	0.005^*^
chemotherapy				
YES vs. NO	1.11 (0.70-1.76)	0.670		
IMRT				
YES vs. NO	0.79 (0.51-1.24)	0.310		
SII				
>403 vs. ≤403	2.34 (1.46-3.74)	<0.001^*^	2.26 (1.40-3.66)	0.001^*a^
NLR				
>2.26 vs. ≤2.26	1.58 (1.04-2.38)	0.031^*^	1.66 (1.08-2.53)	0.020^*b^
PLR				
>112 vs. ≤112	1.54 (1.01-2.35)	0.047^*^	1.54 (0.99-2.38)	0.051^c^
MLR				
>0.25 vs. ≤0.25	1.98 (1.17-3.35)	0.011^*^	1.99 (1.17-3.39)	0.011^*d^

BMI: body mass index; IMRT: intensity-modulated radiotherapy; SII: systemic immune-inflammation index; PLR: platelet lymphocyte ratio; NLR: neutrophil lymphocyte ratio; MLR: monocyte lymphocyte ratio; HR: hazard ratio; CI: confidence interval; Ref: reference. ^a^ The variables (sex, age, T stage, N stage and SII) were tested in a multivariate analysis. ^b^ The variables (sex, age, T stage, N stage and NLR) were tested in a multivariate analysis. ^c^ The variables (sex, age, T stage, N stag and PLR) were tested in a multivariate analysis. ^d^ The variables (sex, age, T stage, N stag and MLR) were tested in a multivariate analysis.

**Figure 2:**
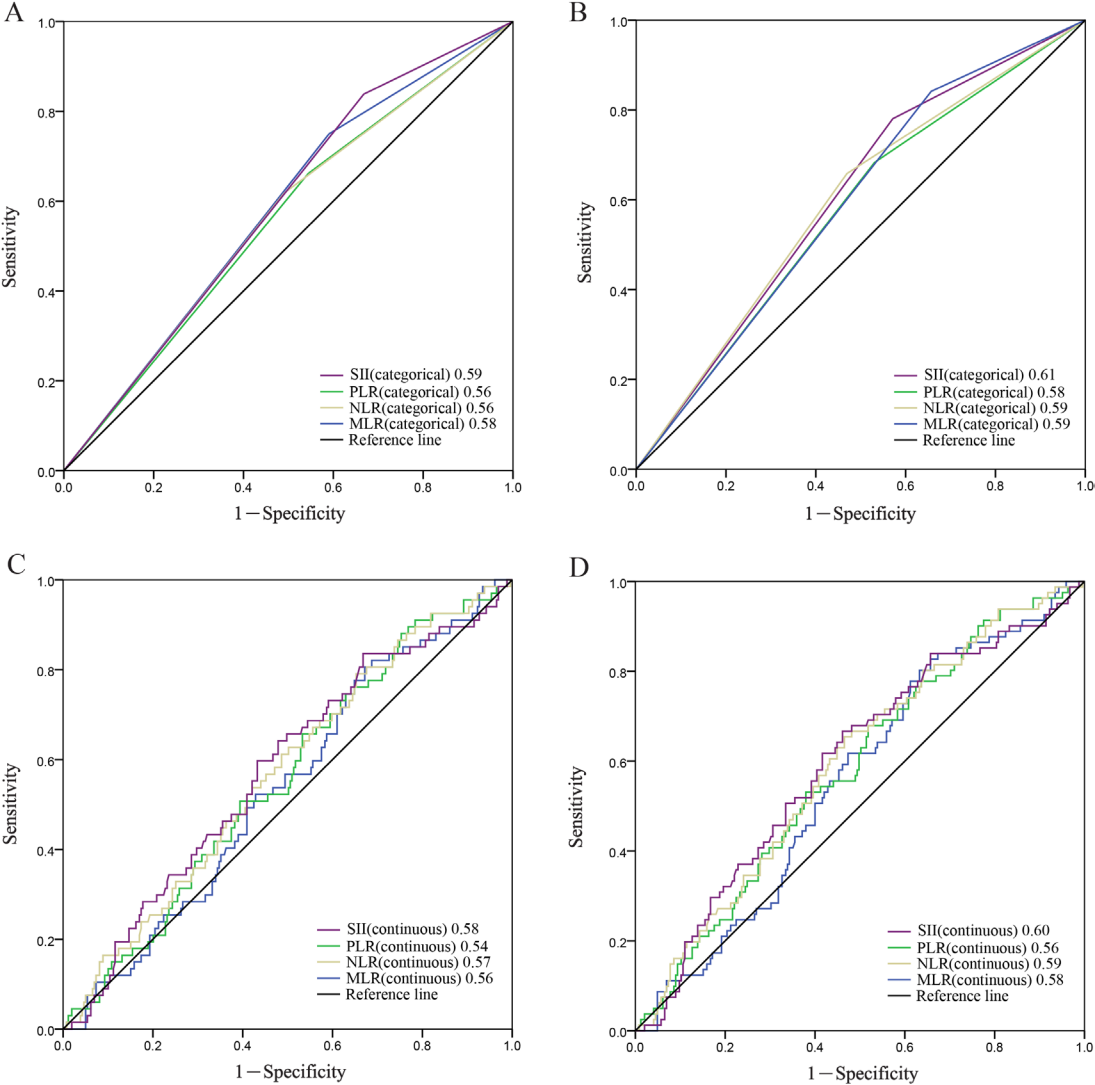
Predictive ability of the SII (categorical) was compared with PLR(categorical), NLR(categorical) and MLR(categorical) by ROC curves in 3-years **(A)** and 5-years **(B)**. Predictive ability of the SII (continuous) was compared with PLR(continuous), NLR(continuous) and MLR(continuous) by ROC curves in 3-years **(C)** and 5-years **(D)**.

To compare the discriminatory ability of SII, PLR, NLR, MLR, we generated ROC curves for the survival status at 3 year and 5 years of follow-up and statistically compared the differences of estimated AUC (Figure 2). The results showed that at the follow-up of 3 year, the AUC value of SII (categorical) was significantly higher than that of the NLR, PLR or MLR. The AUC for SII (categorical) was still higher than NLR, PLR and MLR for predicting survival in patients with NPC in 5-years (Figure 2A, 2B). SII (continuous) showed the same results (Figure 2C, 2D). It indicated that SII is superior to NLR, PLR or MLR as a predictive biomarker in NPC patients.

**Figure 3:**
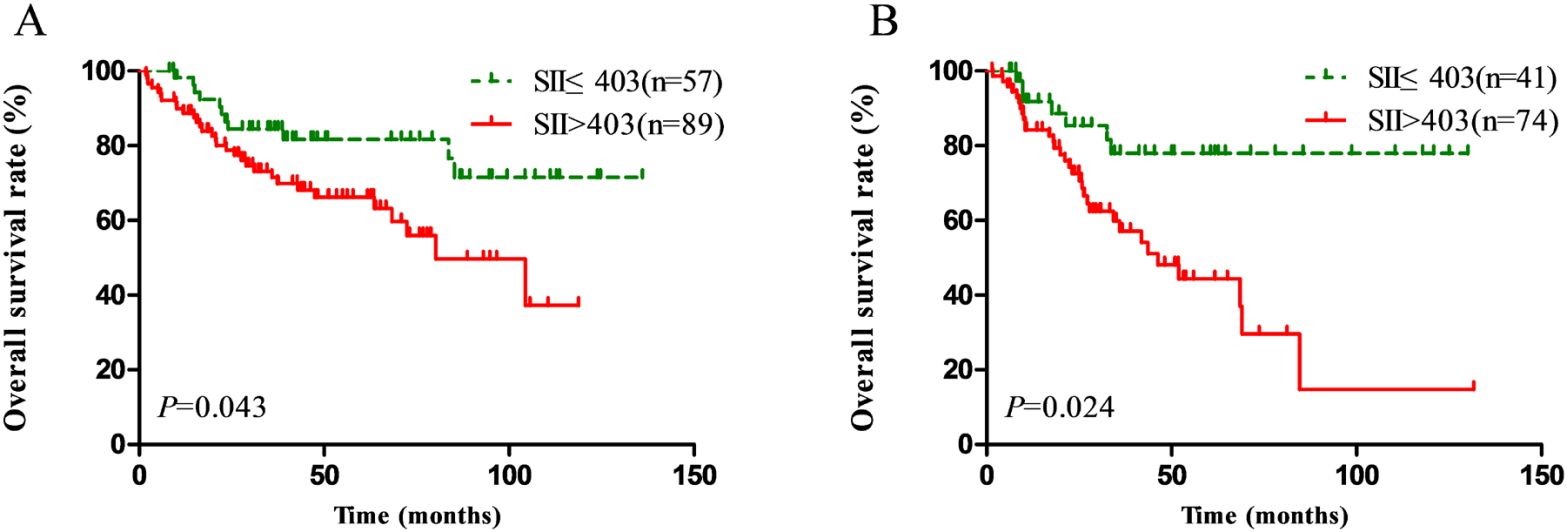
Effect of the SII on the survival of nasopharyngeal carcinoma patients in III stage **(A)**, and IV stage **(B).**

In order to further identify features of patients with better value of SII in the different TNM staging, we performed subgroup survival analysis. It included only the cases with III and IV disease for subgroup analysis because the patient number in I and II stage was small. We observed that in III and IV patients, high SII scores was significantly associated with poor OS (*P*=0.043 for III patients and *P*=0.024 for IV patients) as shown in Figure 3A and 3B.

### Propensity score matching analysis

**Figure 4:**
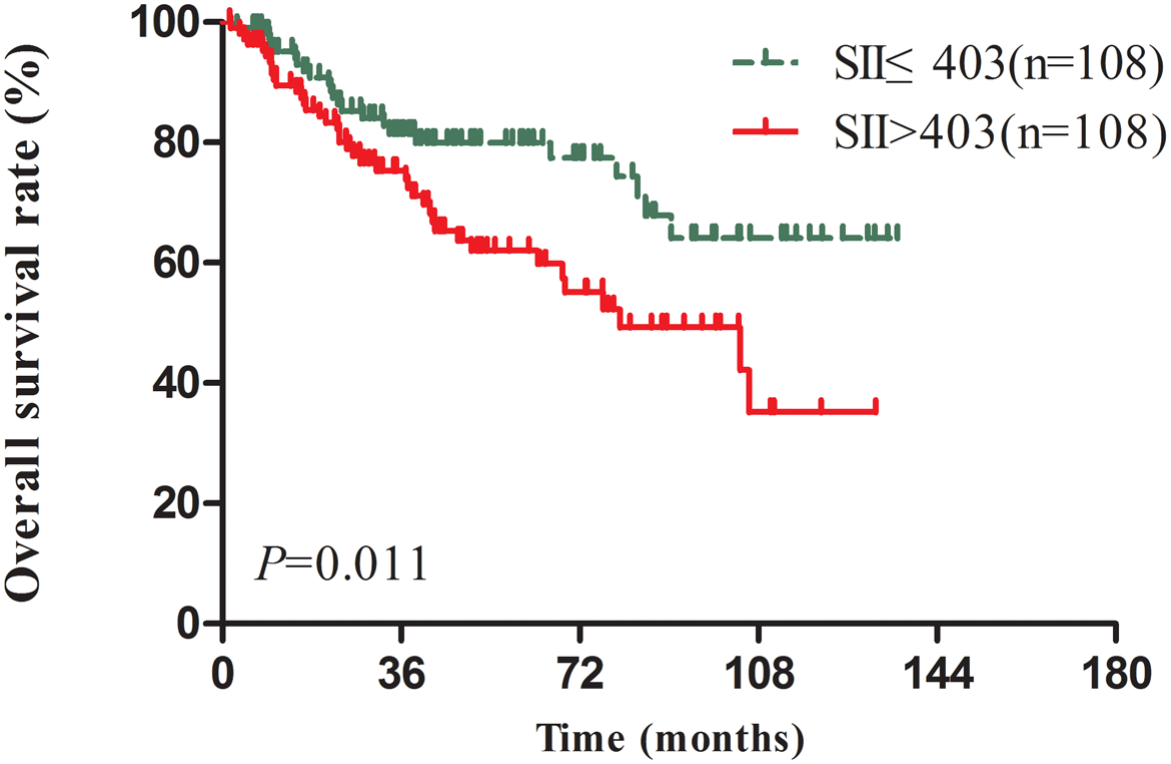
Kaplan–Meier-estimated overall survival distributions from matched datasets for SII≤403 versus SII>403

Considered the BMI, T stage and AJCC TNM stage were imbalance between SII≤403 and SII>403 NPC patients which may affect the reliability of the results (Table 1), we applied a 1:1 PSM ratio to minimize these differences. In the PSM analysis, we selected 108 patients from SII≤403 group with matched pairings of the 108 SII>403 patients. Sex, age, BMI, T stage, N stage and AJCC stage were included for the one-to-one match process. The main characteristics were balanced and evenly distributed between two groups (all *P*>0.2) (Table 1). In the matched 216 patients’ survival analysis, high SII scores was significantly associated with poor OS (*P*=0.011) (Figure 4). In addition, the multivariate analyses indicated SII remained an independent predictor for OS in NPC patients (HR=2.08, CI 1.22-3.55, *P*=0.007) (Table 4).

**Table 4:** Univariate and multivariate cox regression analyses for overall survival in patients with nasopharyngeal carcinoma (matched datasets, 1:1)

Variables	Univariate analysis		Multivariate analysis	
	HR (95%CI)	*P* value	HR (95%CI)	*P* value
Sex				
Female vs. Male	0.49 (0.25-0.93)	0.030^*^	0.38 (0.19-0.74)	0.005^*^
Age				
≥50 vs. <50	1.54 (0.90-2.64)	0.120		
BMI				
>25 vs. ≤25	1.02 (0.59-1.76)	0.944		
T stage		0.013^*^		0.015^*^
T1	Ref.		Ref.	
T2	2.50 (0.99-6.35)	0.053	2.21 (0.85-5.73)	0.103
T3	3.25 (1.30-8.11)	0.012^*^	3.38 (1.33-8.60)	0.011^*^
T4	4.49 (1.78-11.31)	0.001^*^	4.12 (1.62-10.48)	0.003^*^
N stage		0.040^*^		0.066
N0	Ref.		Ref.	
N1	1.52 (0.91-10.10)	0.071	3.08 (0.91-10.51)	0.072
N2	3.57 (1.08-11.79)	0.037^*^	3.57 (1.06-12.00)	0.040^*^
N3	6.59 (1.75-24.83)	0.005^*^	6.12 (1.58-23.64)	0.009^*^
chemotherapy				
YES vs. NO	1.09 (0.62-1.63)	0.774		
IMRT				
YES vs. NO	0.86 (0.49-1.51)	0.608		
SII				
>403 vs. ≤403	1.91 (1.15-3.20)	0.013^*^	2.08 (1.22-3.55)	0.007^*^

BMI: body mass index; IMRT: intensity-modulated radiotherapy; SII: systemic immune-inflammation index; HR: hazard ratio; CI: confidence interval; Ref: reference.

## DISCUSSION

Recently, more and more studies have indicated that inflammation plays an important role in cancer development and metastasis [7]. Pretreatment NLR, PLR and MLR were unfavorable prognostic factors for NPC patients in previous studies [8, 10, 19–22]. A novel systemic inflammation score-SII, as a prognostic factor for poor survival, was considered to be superior to NLR and PLR in hepatocellular carcinoma [11–13], small cell lung cancer [17], esophageal cancer [14] and gastric cancer [16]. However, the prognostic value of SII has not been reported in NPC. In this study, pretreatment SII was confirmed to be a novel independent prognostic factor for patients with NPC. The prognostic value of SII is greater than NLR, PLR and MLR in NPC patients. In addition, SII was significantly correlated with OS in different TNM stage subgroup. Compared with other prognostic factors, SII based on standard laboratory measurements of total platelet, neutrophil, and lymphocyte counts is simple, noninvasive and low cost in clinical practice. Thus, there is a potential for SII to be used as a marker for prognosis and treatment response surveillance.

Several potential mechanisms may explain our results. First, neutrophils, as a type of inflammatory cells, are involved in different steps of tumor development through the production of a variety of cytokines, such as oncostatin M, interleukin-6, hepatocyte growth factor, and tumor necrosis factor [23, 24]. It can enhance the invasion, proliferation, and metastasis of cancer cells as well as aid them to evade immune surveillance [25, 26]. The elevated neutrophils release plenty of reactive oxygen species and nitric oxide. They can lead to T cell activation disorders [27]. Second, platelets can direct contact with circulating tumor cells. It promotes tumor cell extravasation to metastatic sites [28, 29]. In addition, increased circulating platelets and neutrophils produce vascular endothelial growth factor, angiopoietin-1, and fibroblast growth factor-2, causing tumor angiogenesis. [11, 30, 31]. Third, cytotoxic lymphocytes play a fundamental role in cell-mediated immunologic destruction of cancer cells. Circulating lymphocytes can secrete several cytokines, such as IFN-γ and TNF-α, to control tumor growth and improve prognosis of cancer patients [32], and the decreased lymphocyte count and function cannot be responsible for immune surveillance to remove tumor cells [25, 32]. Fourth, Monocytes release monocyte chemoattractant protein-1 to stimulate and mediate tumor-associated monocyte infiltration in solid tumors and then produce various chemokines, such as TGF-α, TNF-α, IL-1, and IL-6 which promote tumorigenesis, angiogenesis, and distant metastasis of malignant tumors [33].

In recent years, several studies showed that systemic inflammatory scores have emerged as prognostic markers in NPC. He et al. reported pretreatment NLR and percentages of lymphocyte and neutrophil are independent prognostic factors and may serve as clinically convenient and useful biomarkers for survival of patients with NPC [19]. Sun et al. reported that pretreatment NLR and PLR can be independent prognostic factors for patients with NPC [20]. Jiang et al. reported elevated PLR values were associated with poor overall survival, cancer-specific survival, and distant metastasis-free survival for patients with NPC [22]. Lu et al. reported the pretreatment NLR was an independent prognostic factor in NPC, and NLR, LMR, and PLR might be a useful complement to TNM staging in the prognostic assessment of NPC patients [21]. In the subsequent meta-analysis, Su et al. found NLR and lymphocyte counts were the 2 most reported prognostic predictors for patients with NPC [10]. However, no study evaluated the prognostic value of SII. SII was based on three circulating immune cells, while NLR, PLR and MLR was based on two circulating immune cells. SII should be a more objective marker that reflects the balance between host inflammatory and immune response status than all the other systemic inflammation scores. In fact, our results confirmed that SII is indeed superior to PLR, NLR and MLR.

Although our results are valuable in NPC, there are some limitations in our study. First, our study was conducted retrospectively in a single center, and the prognostic value of the SII was not verified in a validation cohort. However, we used PSM analysis which can minimize group differences in the baseline characteristics. Second, there was heterogeneity in the patient treatment, thus it was hard to analyze the impact of the SII on patients’ outcome in different treatment patterns. Third, EBV-DNA was one of the important risk factors of distant metastasis. It was not included in this analysis mainly because EBV-DNA was not performed as a routine clinical practice in our hospital before 2008. Forth, due to the absence of some data, we cannot classify all patients according to the 8th AJCC stage. Finally, some diseases such as diabetes, rheumatic diseases and autoimmune disease, may also affect circulating immune cells, which may influence the SII score.

In conclusion, pretreatment SII is a novel independent prognostic predictor for patients with NPC. The prognostic value of SII is superior to PLR, NLR and MLR in NPC. Based on low cost and easy determination of a full blood count, SII will be a potential marker for NPC prognosis and treatment response surveillance.

## MATERIALS AND METHODS

### Patients

From January 2004 to December 2012, newly identified NPC patients who enrolled in the Third Affiliated Hospital of Soochow University were studied. The inclusion criteria were: (1) NPC was confirmed by histopathology, (2) Karnofsky performance score (KPS)≥70, (3) patient had detailed medical records, including MRT, CT, and bone scan for staging, (4) patients received radiotherapy for the first time, (5) patients had measurement of neutrophil, platelet, lymphocyte and monocyte at the same time within 1 weeks before therapy. The exclusion criteria were: (1) distant metastases before or during radiotherapy, (2) radiotherapy uncompleted, (3) any severe coexisting disease mainly including severe dysfunction of heart, lung, liver, or kidney, (4) signs of infection such as acute pancreatitis, cholangitis, or other active concomitant infections. At last, 327 patients were enrolled in this study. This study was undertaken according to the Declaration of Helsinki and was approved by the Ethics Committee of Third Affiliated Hospital of Soochow University. Written informed consent was obtained from all patients.

327 patients were treated with continuously definitive radiotherapy with daily fractions of 2.0 Gy and five fractions per week by 6-8 MV x-ray. Among them, 198 patients with NPC were treated with 2-dimensional radiotherapy (2DRT), and 129 were treated with intensity-modulated radiotherapy (IMRT). The primary tumor was given a total dose of 60–78 Gy in 2DRT. In IMRT, the radiation dose-ranges to the nasopharynx, lymph node-positive area and lymph node-negative area were 60–80, 60–70 and 50–56 Gy, respectively. The regimen of inductive chemotherapy was one or two cycles of paclitaxel (135 mg/m^2^) and nedaplatin (80 mg/m^2^) every 3 weeks. Two to three cycles of nedaplatin (80mg/m^2^) every 3 weeks were used in concurrent chemoradiotherapy. A total of 259 (79.2%) patients received chemotherapy, with 36 patients treated with inductive chemotherapy only, 69 patients treated with concurrent chemotherapy and 154 patients treated with inductive chemotherapy plus concurrent chemotherapy.

All peripheral blood was collected and tested for neutrophils, lymphocytes, platelet, and monocyte counts within 1 weeks before therapy. The definitions of SII, PLR, NLR and MLR are described as follows: SII= platelet*neutrophil/lymphocyte; PLR= platelet/lymphocyte; NLR= neutrophil/lymphocyte; MLR= monocyte/lymphocyte. The optimal cutoff values including SII (SII≤403, SII>403), NLR (NLR≤2.26, NLR>2.26), PLR (PLR≤112, PLR>112) and MLR (MLR≤0.25, MLR>0.25) were determined by using X-tile software (http://www.tissuearray.org/rimmlab) [34].

### Follow-up

All patients were followed up every three months in the first 2 years, every six months until 5 years, and then once annually. The latest follow-up was conducted at the end of August 2015. All patients were followed up by phone calls and regular letters. The observation time in this study was the interval from the date of diagnosis to death or latest follow-up. Survived patients were censored on the day of the last follow-up. The median follow-up was 38.3 months (range, 2 to 164.6 months).

### Statistical analysis

The correlations between SII, PLR, NLR and MLR and clinicopathological factors were analyzed by the χ^2^ test. Pearson correlation analyses were applied to analyze the correlation among SII, PLR, NLR and MLR. Survival curves were plotted using the Kaplan-Meier method and compared using the log-rank test. Univariate and multivariate analyses were calculated by the Cox proportional hazards regression model. The time-dependent receiver operating characteristics (ROC) curve was used to compare the discrimination ability for OS. PSM was carried out because of imbalance in the baseline characteristics. PSM was done with a nearest-neighbour matching algorithm, allowing a maximum tolerated difference between propensity scores less than 30% of the propensity score SD. Standardised group differences were calculated as the means divided by the square root of the half sum of the two variances [35]. PSM was carried out using SPSS 22.0 (SPSS, Chicago, IL) with Statistics Regression and Python Essentials. Statistical analysis was conducted with SPSS 22.0 (SPSS, Chicago, IL), and Graphpad Prism 6.01 (La Jolla, CA, USA). A *P* value less than 0.05 was considered to indicate statistical significance.
